# Reports on the Hospitals of the United Kingdom

**Published:** 1910-05-07

**Authors:** Henry Burdett


### REPORTS
### ON THE
### Hospitals of the United Kingdom.

By SIR HENRY BURDETT, K.C.B., K.C.V.O.

### XLIII.—CHORLTON UNION INFIRMARY, WEST DIDSBURY (MANCHESTER).

This hospital is well situated on an ample site, with abundance of space for extensions. The grounds are considerable, well planted and attractive, and contain ample tennis courts and gardens, so that the staff may obtain good exercise without going outside them. The wards on the whole are good; the pavilions containing them may be roughly classified under three heads: old, intermediate, new. The old wards are not nearly complete, and lack many modern features; the intermediate are good on the whole, the ward units being fair, but incomplete. The new wards are very fair, but the mistake has been made of spending too little money on fittings and decoration, so that they do not offer that increased attractiveness or those facilities which they might and ought to contain. There is no extravagance so unjustifiable on the part of a public body as that which permits the fixing and selection of second-class fittings and appliances in the hospital buildings. Such apparatus never gives satisfaction. It is defective in wearing qualities, and disheartening to the staff from its unsuitability, which produces the appearance of untidiness and neglect in a very short time. We hope that the Chorlton Guardians will take these teachings of experience to heart, and that when the contracts are given out for new pavilions in the future they will take steps to provide that none but the very best fittings and appliances are scheduled in such contracts.

### The Wards.

The walls of the wards are plaster, but on the whole we do not think that they compare favourably with those at Crumpsall. The truth is, too little brains and knowledge are put into the selection of colours, and the scheme of decoration followed is often so crude as to be worthy of children rather than grown men. Good taste and artistic arrangement and selection do not cost extra money any more than the use of a particular colour, yet the avoidable misery and discomfort of ugly and depressing surroundings resulting from want of care in details of this kind are genuinely large, and should be avoided where the management is fired by the ambition to be regarded as efficient.

We imagine that a residence in this infirmary, when it entails remaining in bed for a long period, must involve some and great discomforts to a patient from the fact that the beds are of straw and the pillows of flock. This is the worst type of bedding we have met with in any British hospital devoted to the care of the sick. It is remarkable from the circumstance that the Chorlton Guardians are locally held to be ambitious to make their hospital really efficient, and that the expenditure is large in consequence. We were unable to test the truth or otherwise of this opinion of the public, but it is certainly current in Manchester. In any case, the flock pillows and straw beds should be abolished, and feather pillows and hair mattresses substituted without delay. In fact, this reform would not only add to the comfort of the patients, but prove in practice a real economy.

Several of the ward sculleries and kitchens are too small; they should be reconstructed, and the expense would not be great. The food is sent up from the kitchen in heated tins, and is cut up by the sister in the ward kitchen. This is a good arrangement, and results in a much better and more appetising meal for the patients than any other system. The quality of the food we saw was good, and so was that of the beef-tea and puddings. It would be hard to exaggerate the blessings which the patients have experienced through the introduction of trained nurses, under a capable and zealous superintendent like Miss Smith. We are of opinion, however, that the lady superintendent and her assistants are much underpaid at all these Manchester Poor Law Infirmaries. The lady superintendent ought to receive £250, rising to £300 at least, at all the larger Poor Law Infirmaries, and the salary of her chief assistant should begin at £100 per annum, with a commencing salary of £60 a year for the home sister and junior assistants.

### The Maternity Department.

There is much that is good in the Chorlton Union Poor Law Infirmary, but the best feature in it is undoubtedly this department. In all the circumstances, we do not think that a better administered, more efficient maternity section is to be met with in any Poor Law Infirmary. The sister-in-charge, who has been there seven years, is well up in her work, and has brought her ward and everything connected with it to a high state of perfection. Everything was in perfect order and magnificently clean. The results of the last seven years are extraordinarily good, a fact which we attribute in large measure to the ability and zeal of the sister-in-charge and her assistants. All honour to them.

### The Nurses' Home.

The older home, where the nurses' dining-hall is situated, is not modern, but is sufficient for its purpose. The new home is well equipped, and has many pleasant features. The recreation and sitting rooms for the sister and nurses, with the writing and class rooms, are all good, but we think it reasonable that a greater number of comfortable chairs should be provided for the nurses' use. The recreation-room has a beautiful view from its windows, which look out over the gardens and grounds. It has a polished floor, and is frequently used for dancing. The instruction and training of the probationers are good, and include a cooking class, midwifery, and monthly nursing. The special training in midwifery is confined to those nurses who take the highest places in the examinations, all the other probationers being trained as monthly nurses. We are of opinion that the training of a Poor Law nurse probably fits her better for the duties of a private nurse than any other training open to probationers at the present time.

The **Staffordshire General Infirmary** and **Some Cottage Hospitals** will be reported on next week.

